# Relationship Between Social Vulnerability Index and Severity of Pretreatment Swallowing Dysfunction for Oropharyngeal Cancer Patients

**DOI:** 10.1002/oto2.70242

**Published:** 2026-05-04

**Authors:** Prerita Pandya, Shravan Asthana, Hannah Soltani, Alex Clain, Kirsten B. Burdett, Laila A. Gharzai, Urjeet Patel, Sandeep Samant, Andrew P. Stein, Bonnie Martin‐Harris, Katelyn O. Stepan

**Affiliations:** ^1^ Department of Otolaryngology–Head and Neck Surgery Feinberg School of Medicine, Northwestern University Chicago Illinois USA; ^2^ Roxelyn and Richard Pepper Department of Communication Sciences and Disorders Northwestern University Evanston Illinois USA; ^3^ Division of Biostatistics & Informatics, Department of Preventive Medicine Feinberg School of Medicine, Northwestern University Chicago Illinois USA; ^4^ Department of Radiation Oncology Feinberg School of Medicine, Northwestern University Chicago Illinois USA

**Keywords:** modified barium swallow impairment profile, oropharyngeal squamous cell carcinoma, pretreatment swallowing, social vulnerability index

## Abstract

**Objective:**

Given growing recognition that social determinants of health influence cancer outcomes and functional status, we sought to examine associations between social vulnerability, measured by CDC's Social Vulnerability Index (SVI), and pretreatment swallowing function, quantified by Modified Barium Swallow Impairment Profiles (MBSImP), in patients with oropharyngeal squamous cell carcinoma (OPSCC).

**Study Design and Setting:**

Retrospective analysis of patients at Northwestern University with pretreatment MBSImP studies.

**Methods:**

MBSImP oral total (OT) and pharyngeal total (PT) scores were calculated, and SVI assigned to patient residential address at diagnosis. Associations between overall SVI, SVI subcomponents, clinical variables, and OT and PT scores were assessed using univariable and multivariable linear regression.

**Results:**

149 OPSCC patients with median age 63 years; 83.9% were male, 89.9% White, and 91.9% p16+. On univariable analysis, SVI was significantly associated with higher (worse) OT (*β* = 3.3; 95% CI: 1.8, 4.8; *P* < .001) and PT scores (*β* = 3.6; 95% CI: 0.75, 6.4; *P* = .013). After multivariable adjustment, SVI remained independently associated with increased OT scores (*β* = 2.3; 95% CI: 0.36, 4.2; *P* = .020); PT impairment was independently predicted by tumor subsite (tonsil vs. base of tongue: *β* = −1.8; 95% CI: −3.4, −0.28; *P* = .021). All four SVI domains were significantly linked to OT in univariable models.

**Conclusion:**

Social vulnerability independently predicts oral‐phase swallowing impairment in OPSCC patients, while tumor subsite primarily determines pharyngeal swallowing impairment. These findings highlight the need to consider social determinants in pretreatment assessment, as well as further research to clarify how social determinants impact swallowing outcomes to identify effective, targeted interventions for high‐risk groups.

Socioeconomic status (SES), influenced by factors including education, income, or occupation, has been shown to have important impacts on an individual's health status, access to care, and outcomes.^1^ In head and neck cancer (HNC), including oropharyngeal squamous cell carcinoma (OPSCC), lower SES is associated with more advanced presentation and poorer survival, as well as functional deficits such as dysphagia and social eating.^2^, ^3^, ^4^, ^5^, ^6^ Additionally, swallowing dysfunction is a common and often debilitating condition, contributing considerably to decreased quality of life (QOL) in patients both pretreatment and posttreatment through the influence of factors like age, sex, tumor stage, and treatment type.^7^ In a 2009 study from the Netherland's Cancer Institute, the most frequently encountered pretreatment problems included difficulty swallowing and pain, where 38% of patients with some degree of videofluoroscopic abnormalities.^8^ Dysphagia can also cause psychosocial distress, resulting in weight loss, decreased enjoyment in eating, and meal‐related anxiety, particularly in public spaces.^7^ Among HNC patients, pretreatment dysphagia has been shown to be associated with decreased QOL, feeding tube placement, and decreased survival, while also serving as an independent predictor of posttreatment swallowing dysfunction.^9^, ^10^, ^11^, ^12^ Furthermore, several factors related to SES, such as healthcare access, education, and social determinants of health (SDOH), can influence the severity and progression of dysphagia in OPSCC patients. While dysphagia plays such a pronounced role in the experiences of OPSCC patients, there are minimal data examining the relationship between SES and the degree of pretreatment swallowing impairment among OPSCC patients.

This study aims to investigate the relationship between SES, as determined by social vulnerability index (SVI) score, and physiologic swallowing impairment status, as determined by Modified Barium Swallow Impairment Profiles (MBSImP) scores, in patients with OPSCC. We hypothesized that individuals with higher SVI are more likely to present with greater swallowing impairment.

## Methods

### Data Source

This was a retrospective cohort study involving 239 HNC patients who underwent pretreatment MBSImP studies between 2015 and 2020 at a single academic institution. The patient population and associated variables were gathered from institutional and departmental databases. The study was approved by the Northwestern University Institutional Review Board (IRB IDs: STU00210688 and STU00220891).

### SVI

In this study the Center for Disease Control's (CDC's) SVI was used to quantify an individual's SES due to the variety of variables it includes and the ability to apply it to a larger scale population compared to other measures such as area deprivation index (ADI).^13^ SVI is based on 16 variables from the US Census that are further grouped into 4 categories (household characteristics, racial/ethnicity/language minority status, housing or household type, and transportation) to generate an overall score.^13^ Initially created to identify communities requiring increased and more rapid support in times of disaster, it has more recently been used to assess disparities in health outcomes related to a variety of diseases.^14^, ^15^, ^16^, ^17^, ^18^ For the purposes of this study, SVI was used more generally to denote the extent to which people are likely to be affected by the negative effects of social influences. The SVI scoring system generates a score at the neighborhood and county level ranging from 0.0 to 1.0 with a higher score indicating greater social vulnerability (lower SES).^13^

### Modified Barium Swallow Impairment Profile ™© (MBSImP)

MBSImP quantify swallowing impairment based on videofluoroscopic analysis of 17 validated components of swallowing movements scored on a continuum using Likert scales.^19^ It has been shown to have internal consistency and structural validity, making it clinically relevant and widely used to analyze swallowing impairment.^19^ A modified barium swallow study (MBSS) allows clinicians to visualize a bolus moving through a patient's oral and pharyngeal cavities into their esophagus in real‐time. Scoring is divided into 3 domains: oral, pharyngeal, and esophageal.^20^ Pretreatment MBSImP, performed after diagnosis and before treatment, oral and pharyngeal domain scores were included.

### Inclusion and Exclusion Criteria

**Figure 1:**
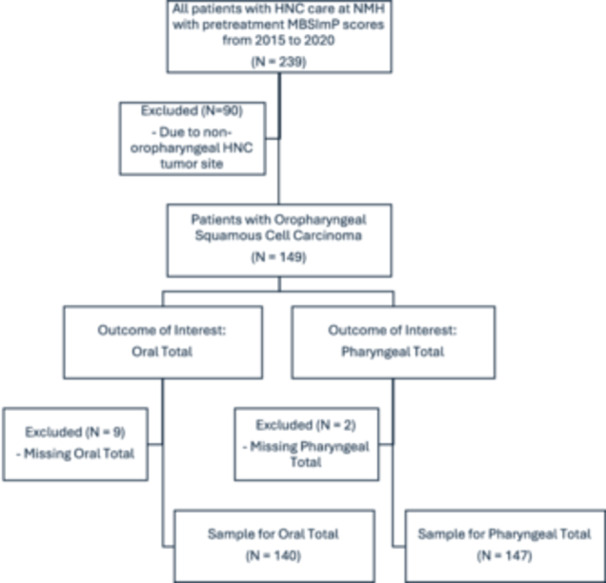
Methodology of obtaining sample for analysis from initial dataset.

Patients with OPSCC were included, resulting in 149 patients. Those missing swallowing impairment outcomes were removed from the analysis (Figure 1).

### Samples

**Figure 2:**
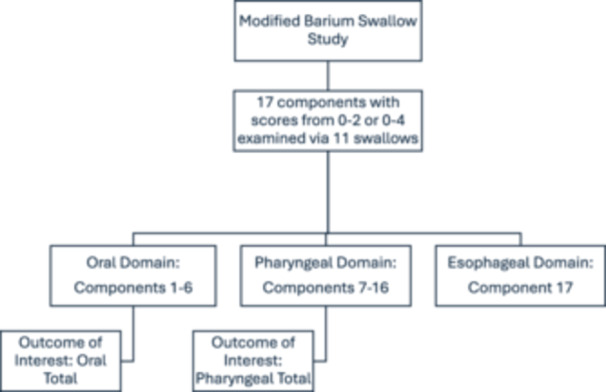
Breakdown of the MBSImP study to obtain OT and PT scores. The chart depicts how the total MBSImP score with 17 independent components was divided into the 3 functional domains (oral, pharyngeal, and esophageal) to obtain the Oral Total and Pharyngeal Total scores from the components associated with the respective domains. OT, oral total; PT, pharyngeal total.

Collected variables included age at diagnosis, sex, race, address at time of diagnosis, alcohol and smoking exposures, tumor stage, tumor subsite, and p16 status. Tumors were staged at diagnosis using American Joint Committee on Cancer (AJCC) 8th edition clinical staging. Due to only 1 patient being T0, T0 was set to NA; T4, T4a, and T4b were all combined into stage T4. Age was changed into 10‐year increments for better interpretation, allowing for the beta‐coefficient to be interpreted in terms of decade per increase. MBSImP oral total (OT) and pharyngeal total (PT) scores were calculated (Figure 2). SVI was measured using patients' residence at time of diagnosis. To obtain SVI scores, patient address was geocoded to the US census Federal Information Processing Standard (FIPS) codes, which generated an SVI score from the CDC. This method was also used to obtain values for the 4 SVI subtypes.

### Statistical Analyses

Descriptive statistics summarized sample characteristics. Continuous variables were reported using median, interquartile range (IQR), minimum, and maximum. Categorical variables were presented as frequencies and percentages. Missing data were tabulated but not included in descriptive analyses. The primary objective was to determine the association between SVI and MBSImP scores. Both OT and PT scores were assessed using linear regression models, before and after adjusting for clinicodemographic variables of interest. Feature selection was done in OT and PT scores separately using stepwise selection with Akaike's Information Criteria (AIC). OT multivariable model adjusted for race, p16 status, and tumor subsite as additive effects. PT multivariable model adjusted for sex, race, p16 status, and tumor subsites as additive effects. Black/African American and Asian race were collapsed due to only 1 patient identifying as Asian. Results included coefficient estimates (*β*) and corresponding 95% confidence intervals (CI). All analyses were conducted at the 0.05 significance level with unadjusted *P*‐values presented. Statistical analyses were performed using R v. 4.2.2 statistical software.

## Results

**Figure 3:**
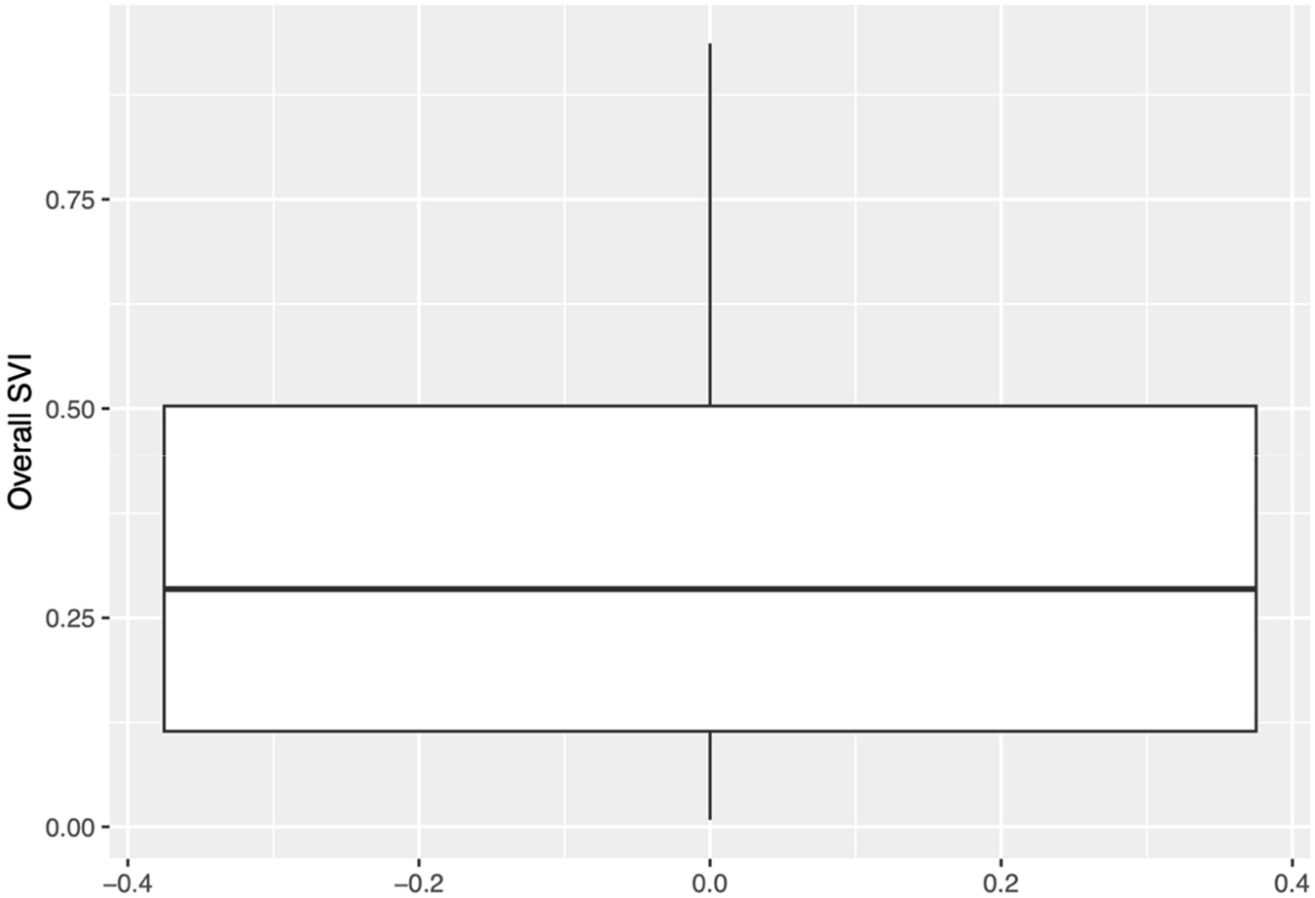
Distribution of overall SVI. The boxplot displays the distribution of the overall SVI scores among the 149 patients. The median SVI score was 0.3, with an IQR of 0.1 to 0.5. The whiskers represent the minimum and maximum observed values from 0.0 to 0.9. SVI, Social Vulnerability Index.

**Figure 4:**
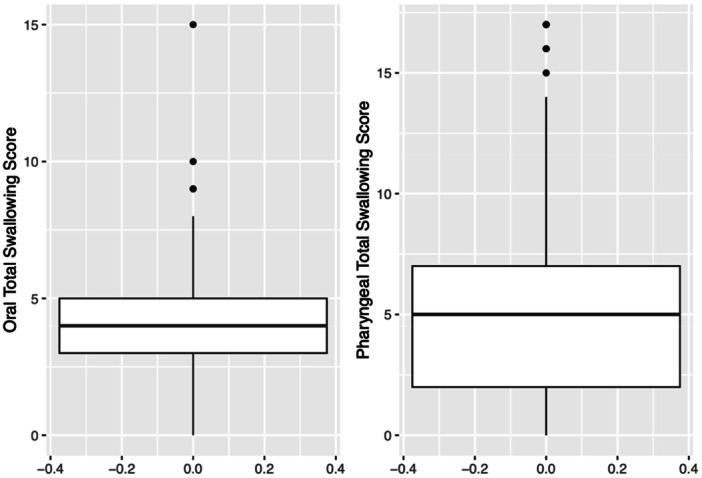
Distribution of OT and PT swallow scores. Boxplots display the distribution of OT (left) and PT (right) swallowing scores. For the OT scores, the observed median was 4, with IQR 3 to 5 and range 0 to 15. Outliers were noted at 9, 10, and 15. For the PT scores, the observed median was 5, with IQR of 2 to 7 and a range of 0 to 17. Outliers were noted at 15, 16, and 17. The whiskers extend to 1.5× the IQR with individual points beyond this range representing outliers. IQR, interquartile range; OT, oral total; PT, pharyngeal total.

Among 149 patients, median age was 63 years (IQR: 55, 68; range 29‐88 years). 83.9% of patients were male; 89.9% identified as White. Most patients were p16 positive (91.9%) (Table 1). The primary site of malignancy involved the tonsil in 69 patients (60%) and base of tongue in 46 patients (40%). 91 patients (61.9%) had stage I disease. 54 patients had T1 staging (37.0%), and 58 patients had T2 staging (39.7%). 89 Patients had N1 staging (60.5%), and 143 patients had M0 staging (97.3%). The distribution of SVI scores (Figure 3) ranged from 0.0 to 0.9 with a median of 0.3 (IQR: 0.1, 0.5). After removing patients with missing MBSImP scores, 140 patients were included in the OT models and 147 patients in the PT models. The distribution of OT scores (Figure 4) ranged from 0 to 15 with a median of 4 (IQR: 3, 5). The distribution of PT scores (Figure 3) ranged from 0 to 17, with a median of 5 (IQR: 2, 7).

**Table 1:** Sample Clinicodemographic Characteristics

Characteristic	N = 149
Sex	
Female	24 (16.1%)
Male	125 (83.9%)
Age at diagnosis (years)	
Median (IQR)	63.0 (55.0, 68.0)
Range	29.0, 88.0
Race	
Black/African American or Asian	15 (10.1%)
White	134 (89.9%)
Ethnicity	
Hispanic/Latino	4 (2.7%)
Non‐Hispanic/Non‐Latino	144 (97.3%)
Missing	1
p16 status	
Negative	12 (8.1%)
Positive	137 (91.9%)
Tumor subsite	
Base of tongue	46 (40.0%)
Tonsil	69 (60.0%)
Missing	34
Tumor stage	
Stage I	91 (61.9%)
Stage II	31 (21.1%)
Stage III	10 (6.8%)
Stage IV+	15 (10.2%)
Missing	2
Alcohol history	
No	73 (50.3%)
Yes	72 (49.7%)
Missing	4
Smoking history	
No	65 (43.6%)
Yes	84 (56.4%)
SVI	
Median (IQR)	0.3 (0.1, 0.5)
Range	0.0, 0.9
n (%)	

For overall SVI, White race, p16 status, T4 stage, and overall stages 3 and 4 were significantly associated with greater social vulnerability (Table 2). When looking at the individual SVI components, White race, T4 stage, and positive smoking history were significantly associated with greater household social vulnerability (Supplemental Table S1, available online). White race, p16 status, T4 stage, and overall stages 3 and 4 were significantly associated with greater race/ethnicity/language SVI scores (Supplemental Table S2, available online). The SES component was significantly associated with race, p16 status, T4 stage, and overall stages 3 and 4 (Supplemental Table S3, available online). No clinical variables were significantly associated with the transportation component (Supplemental Table S4, available online).

**Table 2:** Univariable Analysis of Association Between SVI and Clinical Variables (N = 149)

Characteristic	N	Beta	95% CI	*P*‐value
Age at diagnosis (per 10 years)	149	0.01	−0.03, 0.05	.6
Sex				.6
Female	24	—	—	
Male	125	−0.03	−0.13, 0.08	
Race				<.001
Black/African American or Asian	15	—	—	
White	134	−0.26	−0.39, −0.14	
Insurance				.5
Medicare	90	—	—	
Private	55	−0.01	−0.09, 0.08	
Medicaid	4	−0.15	−0.39, 0.09	
p16 status				.005
Negative	12	—	—	
Positive	137	−0.20	−0.34, −0.06	
Tumor subsite				.4
Base of tongue	46	—	—	
Tonsil	69	−0.04	−0.13, 0.05	
T Stage				.001
T1	54	—	—	
T2	58	0.00	−0.08, 0.08	
T3	13	0.00	−0.14, 0.14	
T4	21	0.22	0.10, 0.33	
N Stage				.2
N0	17	—	—	
N1	89	−0.07	−0.19, 0.06	
N2	38	0.02	−0.11, 0.16	
N3	3	−0.01	−0.30, 0.28	
M stage				.9
M1	4	—	—	
MX	143	0.02	−0.21, 0.26	
Tumor stage				.002
Stage I	91	—	—	
Stage II	31	−0.02	−0.12, 0.07	
Stage III	10	0.21	0.06, 0.36	
Stage IV+	15	0.17	0.05, 0.29	
Alcohol history				.6
No	73	—	—	
Yes	72	−0.02	−0.10, 0.06	
Smoking history				.3
No	65	—	—	
Yes	84	0.04	−0.04, 0.12	

Abbreviations: CI, confidence interval; SVI, Social Vulnerability Index.

### Oral Total Results

Higher overall SVI was significantly associated with increased OT scores in the univariable (*β* = 3.3; 95% CI: 1.8, 4.8; *P* < .001) and multivariable setting (*β* = 2.3; 95% CI: 0.36, 4.2; *P* = .020) (Table 2). On univariable analysis, overall stage (*β* = 1.6; 95% CI: 0.42, 2.8; *P* = .036), T4 stage (*β* = 1.7; 95% CI: 0.56, 2.9; *P* = .034), private insurance status (*β* = 0.99; 95% CI: −1.8, −0.21; *P* = .043), White race (*β* = −1.7; 95% CI: −2.9, −0.43; *P* = .009), and p16 positivity (*β* = −2.0; 95% CI: −3.4, −0.73; *P* = .003) were significantly associated with OT scores. None of these variables were associated with differences in OT scores in multivariable modeling (Table 3).

**Table 3:** Univariable and Multivariable Analysis of OT Score (Overall SVI) (N = 140)

	Univariable				Multivariable		
Characteristic	N	Beta	95% CI	*P*‐value	Beta	95% CI	*P*‐value
SVI	140	3.3	1.8, 4.8	<.001	2.3	0.36, 4.2	.020
Age at Diagnosis (Per 10 Years)	140	0.33	−0.07, 0.72	.10			
Sex				.4			
Female	23	—	—				
Male	117	−0.42	−1.4, 0.60				
Race				.009			.171
Black/African American or Asian	14	—	—		—	—	
White	126	−1.7	−2.9, −0.43		−1.0	−2.5, 0.45	
Insurance				.043			
Medicare	86	—	—				
Private	51	−0.99	−1.8, −0.21				
Medicaid	3	−0.93	−3.5, 1.7				
p16 status				.003			.193
Negative	12	—	—		—	—	
Positive	128	−2.0	−3.4, −0.73		−1.1	−2.7, 0.55	
Tumor subsite				.052			.051
Base of tongue	41	—	—		—	—	
Tonsil	65	−0.89	−1.8, 0.01		−0.86	−1.7, 0.00	
T stage				.034			
T1	50	—	—				
T2	56	0.41	−0.43, 1.2				
T3	12	0.80	−0.58, 2.2				
T4	19	1.7	0.56, 2.9				
N stage				.4			
N0	16	—	—				
N1	84	−0.61	−1.8, 0.59				
N2	35	0.10	−1.2, 1.4				
N3	3	0.17	−2.6, 2.9				
M stage				.8			
M1	4	—	—				
MX	134	−0.35	−2.6, 1.9				
Tumor stage				.036			
Stage I	86	—	—				
Stage II	27	0.31	−0.64, 1.3				
Stage III	10	1.2	−0.28, 2.6				
Stage IV+	15	1.6	0.42, 2.8				
Alcohol history				.7			
No	68	—	—				
Yes	68	−0.13	−0.85, 0.58				
Smoking history				.6			
No	61	—	—				
Yes	79	0.18	−0.59, 0.94				
No. Obs.					106		
AIC					470		
*R*²					0.162		

Abbreviation: CI, confidence interval.

For SVI components, the household (*β* = 1.8; 95% CI: 0.41, 3.1; *P* = .011), race/ethnicity/language (*β* = 2.7; 95% CI: 1.1, 4.3; *P* < .001), SES (*β* = 3.1; 95% CI: 1.5, 4.6; *P* < .001), and transportation (*β* = 1.3; 95% CI: 0.02, 2.6; *P* = .047) SVI components were all significantly associated with higher OT scores on univariable analysis, but not on multivariable analysis (Supplemental Tables S5‐8, available online).

### Pharyngeal Total Results

Higher overall SVI was significantly associated with increased PT scores on univariable analysis (*β* = 3.6; 95% CI: 0.75, 6.4; *P* = .013); however, this did not remain significant in the multivariable model (*β* = 0.21; 95% CI: −3.3, 3.7; *P* = .903) (Supplemental Table S1, available online). In the univariable setting, overall stage (*β* = 2.7; 95% CI: 1.1, 4.4; *P* = .008), subsite (*β* = −1.8; 95% CI: −3.3, −0.23; *P* = .025), T3 and T4 staging ([*β* = 3.2; 95% CI: 0.8, 5.6; *P* = .001] and [*β* = 3.1; 95% CI: 1.0, 5.1; *P* = .001], respectively), White race (*β* = −2.4; 95% CI: −4.7, −0.05; *P* = .046) and private insurance (*β* = −1.6; 95% CI: −3.1, −0.25; *P* = .040) were significantly associated with PT scores. However, in the multivariable model, only tumor subsite remained statistically significant (*β* = −1.8; 95% CI: −3.4, −0.28; *P* = .021), where the tonsillar subsite was associated with lower PT scores (Table 4).

**Table 4:** Univariable and Multivariable Analysis of PT Score (Overall SVI) (N = 147)

	Univariable				Multivariable		
Characteristic	N	Beta	95% CI	*P*‐value	Beta	95% CI	*P*‐value
SVI	147	3.6	0.75, 6.4	.013	0.21	−3.3, 3.7	.903
Age at diagnosis (per 10 years)	147	0.69	−0.03, 1.4	.061			
Sex				.11			.358
Female	24	—	—		—	—	
Male	123	1.5	−0.35, 3.3		0.97	−1.1, 3.1	
Race				.046			.108
Black/African American or Asian	14	—	—		—	—	
White	133	−2.4	−4.7, −0.05		−2.2	−5.0, 0.50	
Insurance				.040			
Medicare	88	—	—				
Private	55	−1.6	−3.1, −0.24				
Medicaid	4	−3.0	−7.2, 1.2				
p16 Status				.093			.199
Negative	12	—	—		—	—	
Positive	135	−2.1	−4.6, 0.36		−2.0	−5.1, 1.1	
Tumor subsite				.025			.021
Base of tongue	45	—	—		—	—	
Tonsil	68	−1.8	−3.3, −0.23		−1.8	−3.4, −0.28	
T stage				.001			
T1	53	—	—				
T2	58	−0.21	−1.7, 1.3				
T3	13	3.2	0.80, 5.6				
T4	20	3.1	1.0, 5.1				
N stage				.052			
N0	17	—	—				
N1	88	−1.2	−3.3, 0.96				
N2	37	0.67	−1.7, 3.0				
N3	3	3.0	−2.1, 8.0				
M stage				0.9			
M1	4	—	—				
MX	141	−0.29	−4.5, 3.9				
Tumor stage				.008			
Stage I	90	—	—				
Stage II	30	2.7	1.1, 4.4				
Stage III	10	2.1	−0.58, 4.7				
Stage IV+	15	1.8	−0.41, 4.0				
Alcohol history				>.9			
No	72	—	—				
Yes	71	0.03	−1.3, 1.4				
Smoking history				.3			
No	64	—	—				
Yes	83	−0.70	−2.1, 0.68				
No. Obs.					113		
AIC					644		
*R*²					0.091		

Abbreviations: CI, confidence interval; SVI, Social Vulnerability Index.

For SVI components, the race/ethnicity/language (*β* = 3.1; 95% CI: 0.18, 6.1; *P* = .038) and SES (*β* = 4.1; 95% CI: 1.3, 7.0; *P* = .005) SVI components were significantly associated with PT scores on univariable analysis, but not multivariable. The household and transportation components were not significantly associated with PT scores on either univariable or multivariable analysis (Supplemental Tables S9‐12, available online).

## Discussion

This study explored the relationship between social vulnerability and pretreatment physiological swallowing impairment in patients with OPSCC, using MBSImP to quantify dysfunction. We found greater social vulnerability, as measured by SVI, is independently associated with greater oral swallowing impairment (OT score), but not pharyngeal impairment (PT score), on multivariable analysis. Additionally, tumor subsite had differential effects, with tongue base tumors causing greater pharyngeal impairment than tonsillar tumors.

On univariable analysis, higher SVI was associated with greater impairment in both the oral and pharyngeal domains. However, after adjusting for clinical variables, SVI remained independently associated only with oral phase dysfunction. Notably, all 4 SVI components—SES, household composition, race/ethnicity/language, and transportation—were significantly linked to oral phase impairment, while only 2 (SES and race/ethnicity/language) were associated with pharyngeal dysfunction. These patterns suggest social vulnerability may affect swallowing function through multiple overlapping pathways, with differential effects across swallowing domains.

Many of the clinical variables adjusted for—p16 status, tumor stage, race, sex, and tumor subsite—were themselves associated with SVI, raising the possibility that they may act as mediators or moderators in the relationship between social vulnerability and swallowing impairment. This highlights the need for further research into the mechanisms by which social determinants influence disease phenotype and functional status, particularly in the context of HNC. Within the OT and PT models, specific variables were adjusted for, as effects, as described in the methods section.

The association between higher SVI and increased OT scores, but not PT scores, may reflect the complex interplay between socioeconomic determinants and neuromuscular control of the oral swallowing. Oral swallowing includes bolus preparation, chewing, and manipulation of the bolus which are voluntary and can be interrupted at any point.^21^ They rely on volitional motor control, coordination, and adequate dentition, all of which can be impacted by factors linked to socioeconomic disadvantage, such as poor oral health, limited access to dental care, and nutritional deficiencies.^21^, ^22^, ^23^, ^24^ In contrast, the pharyngeal swallowing is initiated in response to multiple sensory inputs, relatively “hard wired,” and centrally mediated. Once initiated, pharyngeal components of swallowing become irreversible, proceeding in a fixed synergy, all of which may make it less susceptible to these external social determinants.^21^

Furthermore, individuals in higher SVI strata may have greater baseline oral dysfunction due to chronic comorbid conditions such as diabetes, poor oral hygiene, substance use, and malnutrition—all of which disproportionately affect socially vulnerable populations.^24^, ^25^, ^26^, ^27^, ^28^ These conditions impair tongue strength, bolus formation, and oral transit, directly influencing the OT scores.^29^, ^30^, ^31^, ^32^ Meanwhile, structural and neurologic damage from tumor biology or treatment may dominate pharyngeal impairment, attenuating the observed influence of social factors on PT scores after adjusting for clinical variables.

The differential impact of tumor subsite on swallowing impairment in OPSCC is well established. Numerous studies utilizing both objective (such as the MBSImP and videofluoroscopy) and patient‐reported outcome measures have demonstrated that tongue base tumors are more commonly associated with dysphagia both pretreatment and posttreatment.^33^, ^34^, ^35^, ^36^, ^37^ It has also been shown that among patients with advanced stage oropharyngeal tumors, patients with tongue base primaries had a significantly higher rate of eventual gastrotomy tube dependence.^38^ Anatomically, these tumors often involve the base of the tongue musculature and vallecular space, both critical structures for initiating and coordinating pharyngeal swallowing components and airway protection by way of bolus propulsion from the oral cavity into the pharynx, shielding the laryngeal inlet, and epiglottic movement or tilt, respectively.^34^ Their involvement can impair pharyngeal constriction, pharyngeal stripping, retraction of the tongue base, hyolaryngeal elevation, epiglottic movement, and upper esophageal sphincter opening—all components assessed in the MBSImP PT score, thereby increasing pharyngeal swallowing impairment. In contrast, tonsillar tumors, which are more often laterally situated and confined to the oropharyngeal wall, may exert less direct impact on the musculature central to pharyngeal mechanics, thereby resulting in a less significant effect on the pharyngeal components. Patient‐reported QOL and swallowing function are also better in tonsil cancer survivors compared to those with tongue base primaries.^39^ This anatomical distinction likely explains the stronger association between the tongue base subsite and increased PT scores, and the only marginal association with OT scores, as the oral swallowing structures may remain relatively spared in such cases.

We found more advanced tumor stage to be significantly associated with greater swallow impairment in both oral (*P* = .036) and pharyngeal domains (*P* = .008) on univariable analysis. Tumor staging revealed similar results, with T4 staging significantly associated with worse swallow in both, while T3 was associated only with worse pharyngeal impairment. However, tumor stage was not independently associated with swallow outcomes on multivariable analysis. This finding is consistent with literature that shows tumor stage contributes to pretreatment dysphagia.^40^, ^41^, ^42^ Dysphagia can be related to several factors including tumor bulk, leading to mechanical obstruction and impaired bolus passage, infiltration of key underlying musculature, such as the pharyngeal constrictors or extrinsic tongue muscles, which may impair coordinated bolus propulsion, possible cranial nerve involvement (CN IX, X, XII), particularly in advanced cases, as well as tumor associated pain.^25^, ^26^, ^27^ However, the attenuation of its effect in multivariable analysis suggests that tumor stage may overlap or interact with other clinical and social factors, necessitating further investigation.

Among HNC patients it has been shown that pretreatment dysphagia is not only predictive of post‐treatment swallowing impairment, but is also significantly associated with QOL and survival.^9^, ^10^, ^11^ It is therefore critical to better understand the factors associated with swallowing impairment at the time of presentation so that we might identify and mitigate risk factors, more accurately counsel patients regarding expected functional outcomes, and provide early targeted interventions to minimize long‐term morbidity. The American Society of Clinical Oncology (ASCO) and the American Academy of Otolaryngology–Head and Neck Surgery (AAO‐HNS) both recommend baseline swallowing assessment and risk stratification for all patients with OPSCC. These guidelines highlight the importance of a multidisciplinary approach, involving speech‐language pathologists, dieticians, dental oncologists, and other specialists, to ensure comprehensive preoperative evaluation and to inform both counseling and treatment selection.^43^, ^44^ Previous studies have reported dysphagia to be related to age, gender, tumor location and tumor stage.^45^, ^46^, ^47^ However, there is minimal existing literature regarding the impact of SES on swallow function. One recent study by Patterson et al found that among patients with HNC, patients residing in the most deprived areas, compared to the least, self‐reported poorer swallowing function.^6^ This is consistent with our own findings. Furthermore, it has been well established that patients' perceived swallowing function does not necessarily align with clinical assessment.^45^ This is an area in need of further investigation, and to our knowledge this is the first study to evaluate the impact of SES on objective pretreatment swallow function in OPSCC patients.

Our study is not without limitations. While SVI offers a validated and widely used indicator of neighborhood‐level social vulnerability, capturing a variety of socioeconomic and demographic factors, it does not account for all aspects of SES, such as insurance status, that may impact disease presentation and outcomes.^48^, ^49^ Additionally, this study included a relatively small population size of 149 patients with OPSCC with addresses primarily concentrated in a single metropolitan area, which may limit the generalizability of our findings. The retrospective nature of this analysis introduces potential sources of bias, including incomplete documentation and unmeasured confounders. Moreover, the use of SVI was based on the patients' address at the time of diagnosis, not considering previous addresses that may have impacted social vulnerability prior to diagnosis. As such, our use of SVI provided a limited picture of the effect of a patient's environment and its potential effect on health outcomes. Furthermore, the overall SVI for patients studied was relatively low with a median of 0.3, likely reflecting the characteristics of the source population, which reduces our ability to capture more nuanced associations among patients in higher SVI quartiles.

## Conclusion

Elevated social vulnerability was independently associated with worsened pretreatment oral swallowing impairment in OPSCC patients, and tongue base tumors conferred additional pharyngeal impairment risk. While these findings highlight the potential of SVI to inform risk stratification and identify at‐risk populations in oropharyngeal cancer, integration of SVI into pretreatment clinical decision‐making is not yet established. Future research should elucidate factors mediating the SVI–swallowing relationship, evaluate targeted prehabilitation strategies, and further explore the influence of tumor subsite and socioeconomic determinants on objective and patient‐reported swallow outcomes to inform equitable, personalized care pathways in oropharyngeal cancer.

## Author Contributions

**Prerita Pandya**, BS, conceptualization, methodology, investigation, writing—original draft, visualization; **Shravan Asthana**, BS, conceptualization, methodology, investigation, writing—original draft, visualization; **Hannah Soltani**, BS, conceptualization, methodology, investigation; **Alex Clain**, PhD, methodology, investigation, software, formal analysis, writing—original draft, visualization; **Kirsten B. Burdett**, MS, software, formal analysis, writing—original draft; **Laila Gharzai**, MD, investigation, resources, writing—review and editing; **Urjeet Patel**, MD, investigation, resources; **Sandeep Samant**, MD, investigation, resources; **Andrew P. Stein**, MD, conceptualization, methodology, writing—review and editing; **Bonnie Martin‐Harris**, PhD, conceptualization, methodology, investigation, resources, writing—review and editing, supervision; **Katelyn O. Stepan**, MD, conceptualization, methodology, resources, writing—original draft, supervision.

## Disclosures

### Competing interests

None.

### Funding source

None.

## Supporting information


**Supplemental Table 1:** Univariable analysis of association between household SVI and clinical variables (N = 149).
**Supplemental Table 2:** Univariable analysis of association between race/ethnicity/language SVI and clinical variables (N = 149).
**Supplemental** Table 3**:** Univariable analysis of association between socioeconomic status SVI and clinical variables (N = 149).
**Supplemental** Table 4**:** Univariable analysis of association between Transportation status SVI and clinical variables (N = 149).
**Supplemental Table 5:** Univariable and multivariable analysis of OT score (Household SVI) (N = 140).
**Supplemental Table 6:** Univariable and multivariable analysis of OT score (Race/ethnicity/language SVI) (N = 140).
**Supplemental Table 7:** Univariable and multivariable analysis of OT score (Socioeconomic Status SVI) (N = 140).
**Supplemental Table 8:** Univariable and multivariable analysis of OT score (Transportation SVI) (N = 140).
**Supplemental Table 9:** Univariable and multivariable analysis of PT score (Household SVI) (N = 147).
**Supplemental Table 10:** Univariable and multivariable analysis of PT score (Race/ethnicity/language SVI) (N = 147).
**Supplemental Table 11:** Univariable and multivariable analysis of PT score (Socioeconomic Status SVI) (N = 147).
**Supplemental Table 12:** Univariable and multivariable analysis of PT score (Transportation SVI) (N = 147).
