# Supplementary material for: Relationship Between Social Vulnerability Index and Severity of Pretreatment Swallowing Dysfunction for Oropharyngeal Cancer Patients
Source: OTO Open. 2026 May 4;10(2):e70242. doi: 10.1002/oto2.70242 (PMC13137940; doi:10.1002/oto2.70242)
Supplement: Supplementary file 1 — Supplemental Table 1: Univariable analysis of association between household SVI and clinical variables (N = 149). Supplemental Table 2: Univariable analysis of association between race/ethnicity/language SVI and clinical variables (N = 149). Supplemental Table 3: Univariable analysis of association between socioeconomic status SVI and clinical variables (N = 149). Supplemental Table 4: Univariable analysis of association between Transportation status SVI and clinical variables (N = 149). Supplemental Table 5: Univariable and multivariable analysis of OT score (Household SVI) (N = 140). Supplemental Table 6: Univariable and multivariable analysis of OT score (Race/ethnicity/language SVI) (N = 140). Supplemental Table 7: Univariable and multivariable analysis of OT score (Socioeconomic Status SVI) (N = 140). Supplemental Table 8: Univariable and multivariable analysis of OT score (Transportation SVI) (N = 140). Supplemental Table 9: Univariable and multivariable analysis of PT score (Household SVI) (N = 147). Supplemental Table 10: Univariable and multivariable analysis of PT score (Race/ethnicity/language SVI) (N = 147). Supplemental Table 11: Univariable and multivariable analysis of PT score (Socioeconomic Status SVI) (N = 147). Supplemental Table 12: Univariable and multivariable analysis of PT score (Transportation SVI) (N = 147). [file OTO2-10-e70242-s001.docx]

**Supplemental Table 1:** Univariable analysis of association between household SVI and clinical variables (N=149)

| **Characteristic** | **N** | **Beta** | **95% CI**^1^ | **p-value** |
| --- | --- | --- | --- | --- |
| **Age at Diagnosis (Per 10 Years)** | 149 | 0.03 | -0.02, 0.08 | 0.2 |
| **Sex** |  |  |  | 0.7 |
| Female | 24 | — | — |  |
| Male | 125 | 0.03 | -0.09, 0.15 |  |
| **Race** |  |  |  | 0.009 |
| Black/African American or Asian | 15 | — | — |  |
| White | 134 | -0.19 | -0.33, -0.05 |  |
| **Insurance** |  |  |  | 0.2 |
| Medicare | 90 | — | — |  |
| Private | 55 | -0.01 | -0.10, 0.08 |  |
| Medicaid | 4 | -0.27 | -0.54, 0.00 |  |
| **p16 Status** |  |  |  | 0.2 |
| Negative | 12 | — | — |  |
| Positive | 137 | -0.11 | -0.27, 0.05 |  |
| **Tumor Subsite** |  |  |  | 0.8 |
| Base of Tongue | 46 | — | — |  |
| Tonsil | 69 | -0.01 | -0.11, 0.09 |  |
| **T Stage** |  |  |  | 0.044 |
| T1 | 54 | — | — |  |
| T2 | 58 | 0.00 | -0.10, 0.10 |  |
| T3 | 13 | 0.08 | -0.08, 0.24 |  |
| T4 | 21 | 0.18 | 0.04, 0.31 |  |
| **N Stage** |  |  |  | 0.5 |
| N0 | 17 | — | — |  |
| N1 | 89 | -0.07 | -0.21, 0.08 |  |
| N2 | 38 | 0.00 | -0.15, 0.16 |  |
| N3 | 3 | -0.10 | -0.43, 0.24 |  |
| **M Stage** |  |  |  | 0.3 |
| M1 | 4 | — | — |  |
| MX | 143 | 0.14 | -0.13, 0.41 |  |
| **Tumor Stage** |  |  |  | 0.4 |
| Stage I | 91 | — | — |  |
| Stage II | 31 | 0.00 | -0.11, 0.11 |  |
| Stage III | 10 | 0.14 | -0.03, 0.32 |  |
| Stage IV+ | 15 | 0.03 | -0.12, 0.18 |  |
| **Alcohol history** |  |  |  | 0.2 |
| No | 73 | — | — |  |
| Yes | 72 | 0.06 | -0.03, 0.15 |  |
| **Smoking history** |  |  |  | 0.020 |
| No | 65 | — | — |  |
| Yes | 84 | 0.10 | 0.02, 0.19 |  |
| ^1^CI = Confidence Interval | | | | |

**Supplemental Table 2:** Univariable analysis of association between race/ethnicity/language SVI and clinical variables (N=149)

| **Characteristic** | **N** | **Beta** | **95% CI**^1^ | **p-value** |
| --- | --- | --- | --- | --- |
| **Age at Diagnosis (Per 10 Years)** | 149 | 0.00 | -0.04, 0.04 | >0.9 |
| **Sex** |  |  |  | 0.5 |
| Female | 24 | — | — |  |
| Male | 125 | -0.04 | -0.14, 0.06 |  |
| **Race** |  |  |  | <0.001 |
| Black/African American or Asian | 15 | — | — |  |
| White | 134 | -0.41 | -0.51, -0.30 |  |
| **Insurance** |  |  |  | 0.8 |
| Medicare | 90 | — | — |  |
| Private | 55 | 0.00 | -0.08, 0.08 |  |
| Medicaid | 4 | -0.09 | -0.32, 0.15 |  |
| **p16 Status** |  |  |  | 0.017 |
| Negative | 12 | — | — |  |
| Positive | 137 | -0.17 | -0.30, -0.03 |  |
| **Tumor Subsite** |  |  |  | >0.9 |
| Base of Tongue | 46 | — | — |  |
| Tonsil | 69 | 0.00 | -0.09, 0.09 |  |
| **T Stage** |  |  |  | <0.001 |
| T1 | 54 | — | — |  |
| T2 | 58 | -0.04 | -0.12, 0.04 |  |
| T3 | 13 | -0.01 | -0.14, 0.13 |  |
| T4 | 21 | 0.20 | 0.09, 0.31 |  |
| **N Stage** |  |  |  | 0.005 |
| N0 | 17 | — | — |  |
| N1 | 89 | -0.05 | -0.16, 0.07 |  |
| N2 | 38 | 0.09 | -0.03, 0.22 |  |
| N3 | 3 | 0.21 | -0.06, 0.48 |  |
| **M Stage** |  |  |  | 0.9 |
| M1 | 4 | — | — |  |
| MX | 143 | -0.02 | -0.25, 0.21 |  |
| **Tumor Stage** |  |  |  | 0.019 |
| Stage I | 91 | — | — |  |
| Stage II | 31 | 0.05 | -0.04, 0.14 |  |
| Stage III | 10 | 0.16 | 0.02, 0.31 |  |
| Stage IV+ | 15 | 0.16 | 0.04, 0.28 |  |
| **Alcohol history** |  |  |  | 0.3 |
| No | 73 | — | — |  |
| Yes | 72 | -0.04 | -0.12, 0.04 |  |
| **Smoking history** |  |  |  | 0.7 |
| No | 65 | — | — |  |
| Yes | 84 | -0.01 | -0.09, 0.06 |  |
| ^1^CI = Confidence Interval | | | | |

**Supplemental Table 3:** Univariable analysis of association between socioeconomic status SVI and clinical variables (N=149)

| **Characteristic** | **N** | **Beta** | **95% CI**^1^ | **p-value** |
| --- | --- | --- | --- | --- |
| **Age at Diagnosis (Per 10 Years)** | 149 | 0.00 | -0.04, 0.04 | 0.8 |
| **Sex** |  |  |  | 0.5 |
| Female | 24 | — | — |  |
| Male | 125 | -0.04 | -0.14, 0.06 |  |
| **Race** |  |  |  | <0.001 |
| Black/African American or Asian | 15 | — | — |  |
| White | 134 | -0.25 | -0.37, -0.13 |  |
| **Insurance** |  |  |  | 0.8 |
| Medicare | 90 | — | — |  |
| Private | 55 | -0.01 | -0.08, 0.07 |  |
| Medicaid | 4 | -0.07 | -0.31, 0.17 |  |
| **p16 Status** |  |  |  | 0.003 |
| Negative | 12 | — | — |  |
| Positive | 137 | -0.21 | -0.34, -0.08 |  |
| **Tumor Subsite** |  |  |  | 0.2 |
| Base of Tongue | 46 | — | — |  |
| Tonsil | 69 | -0.06 | -0.14, 0.03 |  |
| **T Stage** |  |  |  | <0.001 |
| T1 | 54 | — | — |  |
| T2 | 58 | -0.03 | -0.11, 0.06 |  |
| T3 | 13 | -0.04 | -0.18, 0.09 |  |
| T4 | 21 | 0.20 | 0.09, 0.31 |  |
| **N Stage** |  |  |  | 0.081 |
| N0 | 17 | — | — |  |
| N1 | 89 | -0.05 | -0.17, 0.07 |  |
| N2 | 38 | 0.06 | -0.07, 0.19 |  |
| N3 | 3 | 0.12 | -0.16, 0.40 |  |
| **M Stage** |  |  |  | 0.8 |
| M1 | 4 | — | — |  |
| MX | 143 | -0.03 | -0.26, 0.21 |  |
| **Tumor Stage** |  |  |  | 0.001 |
| Stage I | 91 | — | — |  |
| Stage II | 31 | -0.01 | -0.10, 0.08 |  |
| Stage III | 10 | 0.23 | 0.09, 0.38 |  |
| Stage IV+ | 15 | 0.17 | 0.05, 0.29 |  |
| **Alcohol history** |  |  |  | 0.12 |
| No | 73 | — | — |  |
| Yes | 72 | -0.06 | -0.14, 0.02 |  |
| **Smoking history** |  |  |  | 0.5 |
| No | 65 | — | — |  |
| Yes | 84 | 0.03 | -0.05, 0.10 |  |
| ^1^CI = Confidence Interval | | | | |

**Supplemental Table 4:** Univariable analysis of association between Transportation status SVI and clinical variables (N=149)

| **Characteristic** | **N** | **Beta** | **95% CI**^1^ | **p-value** |
| --- | --- | --- | --- | --- |
| **Age at Diagnosis (Per 10 Years)** | 149 | 0.03 | -0.02, 0.08 | 0.2 |
| **Sex** |  |  |  | 0.3 |
| Female | 24 | — | — |  |
| Male | 125 | -0.07 | -0.20, 0.06 |  |
| **Race** |  |  |  | 0.5 |
| Black/African American or Asian | 15 | — | — |  |
| White | 134 | -0.06 | -0.22, 0.10 |  |
| **Insurance** |  |  |  | 0.9 |
| Medicare | 90 | — | — |  |
| Private | 55 | -0.03 | -0.13, 0.07 |  |
| Medicaid | 4 | -0.01 | -0.31, 0.28 |  |
| **p16 Status** |  |  |  | 0.14 |
| Negative | 12 | — | — |  |
| Positive | 137 | -0.13 | -0.30, 0.04 |  |
| **Tumor Subsite** |  |  |  | 0.9 |
| Base of Tongue | 46 | — | — |  |
| Tonsil | 69 | 0.01 | -0.10, 0.12 |  |
| **T Stage** |  |  |  | 0.7 |
| T1 | 54 | — | — |  |
| T2 | 58 | 0.04 | -0.06, 0.15 |  |
| T3 | 13 | -0.05 | -0.23, 0.13 |  |
| T4 | 21 | 0.05 | -0.10, 0.20 |  |
| **N Stage** |  |  |  | 0.7 |
| N0 | 17 | — | — |  |
| N1 | 89 | -0.02 | -0.17, 0.13 |  |
| N2 | 38 | -0.05 | -0.21, 0.12 |  |
| N3 | 3 | -0.19 | -0.55, 0.17 |  |
| **M Stage** |  |  |  | 0.7 |
| M1 | 4 | — | — |  |
| MX | 143 | -0.06 | -0.35, 0.23 |  |
| **Tumor Stage** |  |  |  | 0.053 |
| Stage I | 91 | — | — |  |
| Stage II | 31 | -0.10 | -0.21, 0.02 |  |
| Stage III | 10 | 0.03 | -0.15, 0.22 |  |
| Stage IV+ | 15 | 0.15 | -0.01, 0.31 |  |
| **Alcohol history** |  |  |  | >0.9 |
| No | 73 | — | — |  |
| Yes | 72 | 0.01 | -0.09, 0.10 |  |
| **Smoking history** |  |  |  | 0.7 |
| No | 65 | — | — |  |
| Yes | 84 | 0.02 | -0.07, 0.12 |  |
| ^1^CI = Confidence Interval | | | | |

**Supplemental Table 5:** Univariable and multivariable analysis of OT score (Household SVI) (N=140)

|  | **Univariable** | | | | **Multivariable** | | |
| --- | --- | --- | --- | --- | --- | --- | --- |
| **Characteristic** | **N** | **Beta** | **95% CI**^1^ | **p-value** | **Beta** | **95% CI**^1^ | **p-value** |
| **Household SVI** | 140 | 1.8 | 0.41, 3.1 | 0.011 | 0.66 | -0.93, 2.3 | 0.414 |
| **Age at Diagnosis (Per 10 Years)** | 140 | 0.33 | -0.07, 0.72 | 0.10 |  |  |  |
| **Sex** |  |  |  | 0.4 |  |  |  |
| Female | 23 | — | — |  |  |  |  |
| Male | 117 | -0.42 | -1.4, 0.60 |  |  |  |  |
| **Race** |  |  |  | 0.009 |  |  | 0.037 |
| Black/African American or Asian | 14 | — | — |  | — | — |  |
| White | 126 | -1.7 | -2.9, -0.43 |  | -1.5 | -3.0, -0.09 |  |
| **Insurance** |  |  |  | 0.043 |  |  |  |
| Medicare | 86 | — | — |  |  |  |  |
| Private | 51 | -0.99 | -1.8, -0.21 |  |  |  |  |
| Medicaid | 3 | -0.93 | -3.5, 1.7 |  |  |  |  |
| **p16 Status** |  |  |  | 0.003 |  |  | 0.084 |
| Negative | 12 | — | — |  | — | — |  |
| Positive | 128 | -2.0 | -3.4, -0.73 |  | -1.4 | -3.1, 0.20 |  |
| **Tumor Subsite** |  |  |  | 0.052 |  |  | 0.039 |
| Base of Tongue | 41 | — | — |  | — | — |  |
| Tonsil | 65 | -0.89 | -1.8, 0.01 |  | -0.92 | -1.8, -0.05 |  |
| **T Stage** |  |  |  | 0.034 |  |  |  |
| T1 | 50 | — | — |  |  |  |  |
| T2 | 56 | 0.41 | -0.43, 1.2 |  |  |  |  |
| T3 | 12 | 0.80 | -0.58, 2.2 |  |  |  |  |
| T4 | 19 | 1.7 | 0.56, 2.9 |  |  |  |  |
| **N Stage** |  |  |  | 0.4 |  |  |  |
| N0 | 16 | — | — |  |  |  |  |
| N1 | 84 | -0.61 | -1.8, 0.59 |  |  |  |  |
| N2 | 35 | 0.10 | -1.2, 1.4 |  |  |  |  |
| N3 | 3 | 0.17 | -2.6, 2.9 |  |  |  |  |
| **M Stage** |  |  |  | 0.8 |  |  |  |
| M1 | 4 | — | — |  |  |  |  |
| MX | 134 | -0.35 | -2.6, 1.9 |  |  |  |  |
| **Tumor Stage** |  |  |  | 0.036 |  |  |  |
| Stage I | 86 | — | — |  |  |  |  |
| Stage II | 27 | 0.31 | -0.64, 1.3 |  |  |  |  |
| Stage III | 10 | 1.2 | -0.28, 2.6 |  |  |  |  |
| Stage IV+ | 15 | 1.6 | 0.42, 2.8 |  |  |  |  |
| **Alcohol history** |  |  |  | 0.7 |  |  |  |
| No | 68 | — | — |  |  |  |  |
| Yes | 68 | -0.13 | -0.85, 0.58 |  |  |  |  |
| **Smoking history** |  |  |  | 0.6 |  |  |  |
| No | 61 | — | — |  |  |  |  |
| Yes | 79 | 0.18 | -0.59, 0.94 |  |  |  |  |
| No. Obs. |  |  |  |  | 106 |  |  |
| AIC |  |  |  |  | 475 |  |  |
| R² |  |  |  |  | 0.122 |  |  |
| ^1^CI = Confidence Interval | | | | | | | |

**Supplemental Table 6:** Univariable and multivariable analysis of OT score (Race/ethnicity/language SVI) (N=140)

|  | **Univariable** | | | | **Multivariable** | | |
| --- | --- | --- | --- | --- | --- | --- | --- |
| **Characteristic** | **N** | **Beta** | **95% CI**^1^ | **p-value** | **Beta** | **95% CI**^1^ | **p-value** |
| **Race SVI** | 140 | 2.7 | 1.1, 4.3 | <0.001 | 1.3 | -0.82, 3.5 | 0.221 |
| **Age at Diagnosis (Per 10 Years)** | 140 | 0.33 | -0.07, 0.72 | 0.10 |  |  |  |
| **Sex** |  |  |  | 0.4 |  |  |  |
| Female | 23 | — | — |  |  |  |  |
| Male | 117 | -0.42 | -1.4, 0.60 |  |  |  |  |
| **Race** |  |  |  | 0.009 |  |  | 0.174 |
| Black/African American or Asian | 14 | — | — |  | — | — |  |
| White | 126 | -1.7 | -2.9, -0.43 |  | -1.1 | -2.8, 0.50 |  |
| **Insurance** |  |  |  | 0.043 |  |  |  |
| Medicare | 86 | — | — |  |  |  |  |
| Private | 51 | -0.99 | -1.8, -0.21 |  |  |  |  |
| Medicaid | 3 | -0.93 | -3.5, 1.7 |  |  |  |  |
| **p16 Status** |  |  |  | 0.003 |  |  | 0.131 |
| Negative | 12 | — | — |  | — | — |  |
| Positive | 128 | -2.0 | -3.4, -0.73 |  | -1.3 | -2.9, 0.39 |  |
| **Tumor Subsite** |  |  |  | 0.052 |  |  | 0.043 |
| Base of Tongue | 41 | — | — |  | — | — |  |
| Tonsil | 65 | -0.89 | -1.8, 0.01 |  | -0.90 | -1.8, -0.03 |  |
| **T Stage** |  |  |  | 0.034 |  |  |  |
| T1 | 50 | — | — |  |  |  |  |
| T2 | 56 | 0.41 | -0.43, 1.2 |  |  |  |  |
| T3 | 12 | 0.80 | -0.58, 2.2 |  |  |  |  |
| T4 | 19 | 1.7 | 0.56, 2.9 |  |  |  |  |
| **N Stage** |  |  |  | 0.4 |  |  |  |
| N0 | 16 | — | — |  |  |  |  |
| N1 | 84 | -0.61 | -1.8, 0.59 |  |  |  |  |
| N2 | 35 | 0.10 | -1.2, 1.4 |  |  |  |  |
| N3 | 3 | 0.17 | -2.6, 2.9 |  |  |  |  |
| **M Stage** |  |  |  | 0.8 |  |  |  |
| M1 | 4 | — | — |  |  |  |  |
| MX | 134 | -0.35 | -2.6, 1.9 |  |  |  |  |
| **Tumor Stage** |  |  |  | 0.036 |  |  |  |
| Stage I | 86 | — | — |  |  |  |  |
| Stage II | 27 | 0.31 | -0.64, 1.3 |  |  |  |  |
| Stage III | 10 | 1.2 | -0.28, 2.6 |  |  |  |  |
| Stage IV+ | 15 | 1.6 | 0.42, 2.8 |  |  |  |  |
| **Alcohol history** |  |  |  | 0.7 |  |  |  |
| No | 68 | — | — |  |  |  |  |
| Yes | 68 | -0.13 | -0.85, 0.58 |  |  |  |  |
| **Smoking history** |  |  |  | 0.6 |  |  |  |
| No | 61 | — | — |  |  |  |  |
| Yes | 79 | 0.18 | -0.59, 0.94 |  |  |  |  |
| No. Obs. |  |  |  |  | 106 |  |  |
| AIC |  |  |  |  | 475 |  |  |
| R² |  |  |  |  | 0.129 |  |  |
| ^1^CI = Confidence Interval | | | | | | | |

**Supplemental Table 7:** Univariable and multivariable analysis of OT score (Socioeconomic Status SVI) (N=140)

|  | **Univariable** | | | | **Multivariable** | | |
| --- | --- | --- | --- | --- | --- | --- | --- |
| **Characteristic** | **N** | **Beta** | **95% CI**^1^ | **p-value** | **Beta** | **95% CI**^1^ | **p-value** |
| **Socioeconomics SVI** | 140 | 3.1 | 1.5, 4.6 | <0.001 | 1.9 | -0.03, 3.9 | 0.054 |
| **Age at Diagnosis (Per 10 Years)** | 140 | 0.33 | -0.07, 0.72 | 0.10 |  |  |  |
| **Sex** |  |  |  | 0.4 |  |  |  |
| Female | 23 | — | — |  |  |  |  |
| Male | 117 | -0.42 | -1.4, 0.60 |  |  |  |  |
| **Race** |  |  |  | 0.009 |  |  | 0.105 |
| Black/African American or Asian | 14 | — | — |  | — | — |  |
| White | 126 | -1.7 | -2.9, -0.43 |  | -1.2 | -2.7, 0.25 |  |
| **Insurance** |  |  |  | 0.043 |  |  |  |
| Medicare | 86 | — | — |  |  |  |  |
| Private | 51 | -0.99 | -1.8, -0.21 |  |  |  |  |
| Medicaid | 3 | -0.93 | -3.5, 1.7 |  |  |  |  |
| **p16 Status** |  |  |  | 0.003 |  |  | 0.204 |
| Negative | 12 | — | — |  | — | — |  |
| Positive | 128 | -2.0 | -3.4, -0.73 |  | -1.1 | -2.7, 0.59 |  |
| **Tumor Subsite** |  |  |  | 0.052 |  |  | 0.067 |
| Base of Tongue | 41 | — | — |  | — | — |  |
| Tonsil | 65 | -0.89 | -1.8, 0.01 |  | -0.81 | -1.7, 0.06 |  |
| **T Stage** |  |  |  | 0.034 |  |  |  |
| T1 | 50 | — | — |  |  |  |  |
| T2 | 56 | 0.41 | -0.43, 1.2 |  |  |  |  |
| T3 | 12 | 0.80 | -0.58, 2.2 |  |  |  |  |
| T4 | 19 | 1.7 | 0.56, 2.9 |  |  |  |  |
| **N Stage** |  |  |  | 0.4 |  |  |  |
| N0 | 16 | — | — |  |  |  |  |
| N1 | 84 | -0.61 | -1.8, 0.59 |  |  |  |  |
| N2 | 35 | 0.10 | -1.2, 1.4 |  |  |  |  |
| N3 | 3 | 0.17 | -2.6, 2.9 |  |  |  |  |
| **M Stage** |  |  |  | 0.8 |  |  |  |
| M1 | 4 | — | — |  |  |  |  |
| MX | 134 | -0.35 | -2.6, 1.9 |  |  |  |  |
| **Tumor Stage** |  |  |  | 0.036 |  |  |  |
| Stage I | 86 | — | — |  |  |  |  |
| Stage II | 27 | 0.31 | -0.64, 1.3 |  |  |  |  |
| Stage III | 10 | 1.2 | -0.28, 2.6 |  |  |  |  |
| Stage IV+ | 15 | 1.6 | 0.42, 2.8 |  |  |  |  |
| **Alcohol history** |  |  |  | 0.7 |  |  |  |
| No | 68 | — | — |  |  |  |  |
| Yes | 68 | -0.13 | -0.85, 0.58 |  |  |  |  |
| **Smoking history** |  |  |  | 0.6 |  |  |  |
| No | 61 | — | — |  |  |  |  |
| Yes | 79 | 0.18 | -0.59, 0.94 |  |  |  |  |
| No. Obs. |  |  |  |  | 106 |  |  |
| AIC |  |  |  |  | 472 |  |  |
| R² |  |  |  |  | 0.148 |  |  |
| ^1^CI = Confidence Interval | | | | | | | |

**Supplemental Table 8:** Univariable and multivariable analysis of OT score (Transportation SVI) (N=140)

|  | **Univariable** | | | | **Multivariable** | | |
| --- | --- | --- | --- | --- | --- | --- | --- |
| **Characteristic** | **N** | **Beta** | **95% CI**^1^ | **p-value** | **Beta** | **95% CI**^1^ | **p-value** |
| **Transportation SVI** | 140 | 1.3 | 0.02, 2.6 | 0.047 | 1.2 | -0.28, 2.8 | 0.108 |
| **Age at Diagnosis (Per 10 Years)** | 140 | 0.33 | -0.07, 0.72 | 0.10 |  |  |  |
| **Sex** |  |  |  | 0.4 |  |  |  |
| Female | 23 | — | — |  |  |  |  |
| Male | 117 | -0.42 | -1.4, 0.60 |  |  |  |  |
| **Race** |  |  |  | 0.009 |  |  | 0.038 |
| Black/African American or Asian | 14 | — | — |  | — | — |  |
| White | 126 | -1.7 | -2.9, -0.43 |  | -1.5 | -2.9, -0.08 |  |
| **Insurance** |  |  |  | 0.043 |  |  |  |
| Medicare | 86 | — | — |  |  |  |  |
| Private | 51 | -0.99 | -1.8, -0.21 |  |  |  |  |
| Medicaid | 3 | -0.93 | -3.5, 1.7 |  |  |  |  |
| **p16 Status** |  |  |  | 0.003 |  |  | 0.075 |
| Negative | 12 | — | — |  | — | — |  |
| Positive | 128 | -2.0 | -3.4, -0.73 |  | -1.5 | -3.1, 0.15 |  |
| **Tumor Subsite** |  |  |  | 0.052 |  |  | 0.033 |
| Base of Tongue | 41 | — | — |  | — | — |  |
| Tonsil | 65 | -0.89 | -1.8, 0.01 |  | -0.95 | -1.8, -0.08 |  |
| **T Stage** |  |  |  | 0.034 |  |  |  |
| T1 | 50 | — | — |  |  |  |  |
| T2 | 56 | 0.41 | -0.43, 1.2 |  |  |  |  |
| T3 | 12 | 0.80 | -0.58, 2.2 |  |  |  |  |
| T4 | 19 | 1.7 | 0.56, 2.9 |  |  |  |  |
| **N Stage** |  |  |  | 0.4 |  |  |  |
| N0 | 16 | — | — |  |  |  |  |
| N1 | 84 | -0.61 | -1.8, 0.59 |  |  |  |  |
| N2 | 35 | 0.10 | -1.2, 1.4 |  |  |  |  |
| N3 | 3 | 0.17 | -2.6, 2.9 |  |  |  |  |
| **M Stage** |  |  |  | 0.8 |  |  |  |
| M1 | 4 | — | — |  |  |  |  |
| MX | 134 | -0.35 | -2.6, 1.9 |  |  |  |  |
| **Tumor Stage** |  |  |  | 0.036 |  |  |  |
| Stage I | 86 | — | — |  |  |  |  |
| Stage II | 27 | 0.31 | -0.64, 1.3 |  |  |  |  |
| Stage III | 10 | 1.2 | -0.28, 2.6 |  |  |  |  |
| Stage IV+ | 15 | 1.6 | 0.42, 2.8 |  |  |  |  |
| **Alcohol history** |  |  |  | 0.7 |  |  |  |
| No | 68 | — | — |  |  |  |  |
| Yes | 68 | -0.13 | -0.85, 0.58 |  |  |  |  |
| **Smoking history** |  |  |  | 0.6 |  |  |  |
| No | 61 | — | — |  |  |  |  |
| Yes | 79 | 0.18 | -0.59, 0.94 |  |  |  |  |
| No. Obs. |  |  |  |  | 106 |  |  |
| AIC |  |  |  |  | 473 |  |  |
| R² |  |  |  |  | 0.138 |  |  |
| ^1^CI = Confidence Interval | | | | | | | |

**Supplemental Table 9:** Univariable and multivariable analysis of PT score (Household SVI) (N=147)

|  | **Univariable** | | | | **Multivariable** | | |
| --- | --- | --- | --- | --- | --- | --- | --- |
| **Characteristic** | **N** | **Beta** | **95% CI**^1^ | **p-value** | **Beta** | **95% CI**^1^ | **p-value** |
| **Household SVI** | 147 | 2.2 | -0.28, 4.8 | 0.081 | -0.13 | -3.0, 2.7 | 0.929 |
| **Age at Diagnosis (Per 10 Years)** | 147 | 0.69 | -0.03, 1.4 | 0.061 |  |  |  |
| **Sex** |  |  |  | 0.11 |  |  | 0.362 |
| Female | 24 | — | — |  | — | — |  |
| Male | 123 | 1.5 | -0.35, 3.3 |  | 0.96 | -1.1, 3.0 |  |
| **Race** |  |  |  | 0.046 |  |  | 0.080 |
| Black/African American or Asian | 14 | — | — |  | — | — |  |
| White | 133 | -2.4 | -4.7, -0.05 |  | -2.3 | -4.9, 0.28 |  |
| **Insurance** |  |  |  | 0.040 |  |  |  |
| Medicare | 88 | — | — |  |  |  |  |
| Private | 55 | -1.6 | -3.1, -0.24 |  |  |  |  |
| Medicaid | 4 | -3.0 | -7.2, 1.2 |  |  |  |  |
| **p16 Status** |  |  |  | 0.093 |  |  | 0.180 |
| Negative | 12 | — | — |  | — | — |  |
| Positive | 135 | -2.1 | -4.6, 0.36 |  | -2.1 | -5.1, 0.98 |  |
| **Tumor Subsite** |  |  |  | 0.025 |  |  | 0.020 |
| Base of Tongue | 45 | — | — |  | — | — |  |
| Tonsil | 68 | -1.8 | -3.3, -0.23 |  | -1.8 | -3.4, -0.30 |  |
| **T Stage** |  |  |  | 0.001 |  |  |  |
| T1 | 53 | — | — |  |  |  |  |
| T2 | 58 | -0.21 | -1.7, 1.3 |  |  |  |  |
| T3 | 13 | 3.2 | 0.80, 5.6 |  |  |  |  |
| T4 | 20 | 3.1 | 1.0, 5.1 |  |  |  |  |
| **N Stage** |  |  |  | 0.052 |  |  |  |
| N0 | 17 | — | — |  |  |  |  |
| N1 | 88 | -1.2 | -3.3, 0.96 |  |  |  |  |
| N2 | 37 | 0.67 | -1.7, 3.0 |  |  |  |  |
| N3 | 3 | 3.0 | -2.1, 8.0 |  |  |  |  |
| **M Stage** |  |  |  | 0.9 |  |  |  |
| M1 | 4 | — | — |  |  |  |  |
| MX | 141 | -0.29 | -4.5, 3.9 |  |  |  |  |
| **Tumor Stage** |  |  |  | 0.008 |  |  |  |
| Stage I | 90 | — | — |  |  |  |  |
| Stage II | 30 | 2.7 | 1.1, 4.4 |  |  |  |  |
| Stage III | 10 | 2.1 | -0.58, 4.7 |  |  |  |  |
| Stage IV+ | 15 | 1.8 | -0.41, 4.0 |  |  |  |  |
| **Alcohol history** |  |  |  | >0.9 |  |  |  |
| No | 72 | — | — |  |  |  |  |
| Yes | 71 | 0.03 | -1.3, 1.4 |  |  |  |  |
| **Smoking history** |  |  |  | 0.3 |  |  |  |
| No | 64 | — | — |  |  |  |  |
| Yes | 83 | -0.70 | -2.1, 0.68 |  |  |  |  |
| No. Obs. |  |  |  |  | 113 |  |  |
| AIC |  |  |  |  | 644 |  |  |
| R² |  |  |  |  | 0.091 |  |  |
| ^1^CI = Confidence Interval | | | | | | | |

**Supplemental Table 10:** Univariable and multivariable analysis of PT score (Race/ethnicity/language SVI) (N=147)

|  | **Univariable** | | | | **Multivariable** | | |
| --- | --- | --- | --- | --- | --- | --- | --- |
| **Characteristic** | **N** | **Beta** | **95% CI**^1^ | **p-value** | **Beta** | **95% CI**^1^ | **p-value** |
| **Race SVI** | 147 | 3.1 | 0.18, 6.1 | 0.038 | 0.12 | -3.9, 4.1 | 0.954 |
| **Age at Diagnosis (Per 10 Years)** | 147 | 0.69 | -0.03, 1.4 | 0.061 |  |  |  |
| **Sex** |  |  |  | 0.11 |  |  | 0.360 |
| Female | 24 | — | — |  | — | — |  |
| Male | 123 | 1.5 | -0.35, 3.3 |  | 0.97 | -1.1, 3.1 |  |
| **Race** |  |  |  | 0.046 |  |  | 0.136 |
| Black/African American or Asian | 14 | — | — |  | — | — |  |
| White | 133 | -2.4 | -4.7, -0.05 |  | -2.3 | -5.2, 0.72 |  |
| **Insurance** |  |  |  | 0.040 |  |  |  |
| Medicare | 88 | — | — |  |  |  |  |
| Private | 55 | -1.6 | -3.1, -0.24 |  |  |  |  |
| Medicaid | 4 | -3.0 | -7.2, 1.2 |  |  |  |  |
| **p16 Status** |  |  |  | 0.093 |  |  | 0.196 |
| Negative | 12 | — | — |  | — | — |  |
| Positive | 135 | -2.1 | -4.6, 0.36 |  | -2.0 | -5.2, 1.1 |  |
| **Tumor Subsite** |  |  |  | 0.025 |  |  | 0.020 |
| Base of Tongue | 45 | — | — |  | — | — |  |
| Tonsil | 68 | -1.8 | -3.3, -0.23 |  | -1.8 | -3.4, -0.30 |  |
| **T Stage** |  |  |  | 0.001 |  |  |  |
| T1 | 53 | — | — |  |  |  |  |
| T2 | 58 | -0.21 | -1.7, 1.3 |  |  |  |  |
| T3 | 13 | 3.2 | 0.80, 5.6 |  |  |  |  |
| T4 | 20 | 3.1 | 1.0, 5.1 |  |  |  |  |
| **N Stage** |  |  |  | 0.052 |  |  |  |
| N0 | 17 | — | — |  |  |  |  |
| N1 | 88 | -1.2 | -3.3, 0.96 |  |  |  |  |
| N2 | 37 | 0.67 | -1.7, 3.0 |  |  |  |  |
| N3 | 3 | 3.0 | -2.1, 8.0 |  |  |  |  |
| **M Stage** |  |  |  | 0.9 |  |  |  |
| M1 | 4 | — | — |  |  |  |  |
| MX | 141 | -0.29 | -4.5, 3.9 |  |  |  |  |
| **Tumor Stage** |  |  |  | 0.008 |  |  |  |
| Stage I | 90 | — | — |  |  |  |  |
| Stage II | 30 | 2.7 | 1.1, 4.4 |  |  |  |  |
| Stage III | 10 | 2.1 | -0.58, 4.7 |  |  |  |  |
| Stage IV+ | 15 | 1.8 | -0.41, 4.0 |  |  |  |  |
| **Alcohol history** |  |  |  | >0.9 |  |  |  |
| No | 72 | — | — |  |  |  |  |
| Yes | 71 | 0.03 | -1.3, 1.4 |  |  |  |  |
| **Smoking history** |  |  |  | 0.3 |  |  |  |
| No | 64 | — | — |  |  |  |  |
| Yes | 83 | -0.70 | -2.1, 0.68 |  |  |  |  |
| No. Obs. |  |  |  |  | 113 |  |  |
| AIC |  |  |  |  | 644 |  |  |
| R² |  |  |  |  | 0.091 |  |  |
| ^1^CI = Confidence Interval | | | | | | | |

**Supplemental Table 11:** Univariable and multivariable analysis of PT score (Socioeconomic Status SVI) (N=147)

|  | **Univariable** | | | | **Multivariable** | | |
| --- | --- | --- | --- | --- | --- | --- | --- |
| **Characteristic** | **N** | **Beta** | **95% CI**^1^ | **p-value** | **Beta** | **95% CI**^1^ | **p-value** |
| **Socioeconomics SVI** | 147 | 4.1 | 1.3, 7.0 | 0.005 | 0.69 | -3.0, 4.3 | 0.706 |
| **Age at Diagnosis (Per 10 Years)** | 147 | 0.69 | -0.03, 1.4 | 0.061 |  |  |  |
| **Sex** |  |  |  | 0.11 |  |  | 0.347 |
| Female | 24 | — | — |  | — | — |  |
| Male | 123 | 1.5 | -0.35, 3.3 |  | 0.99 | -1.1, 3.1 |  |
| **Race** |  |  |  | 0.046 |  |  | 0.119 |
| Black/African American or Asian | 14 | — | — |  | — | — |  |
| White | 133 | -2.4 | -4.7, -0.05 |  | -2.1 | -4.8, 0.56 |  |
| **Insurance** |  |  |  | 0.040 |  |  |  |
| Medicare | 88 | — | — |  |  |  |  |
| Private | 55 | -1.6 | -3.1, -0.24 |  |  |  |  |
| Medicaid | 4 | -3.0 | -7.2, 1.2 |  |  |  |  |
| **p16 Status** |  |  |  | 0.093 |  |  | 0.231 |
| Negative | 12 | — | — |  | — | — |  |
| Positive | 135 | -2.1 | -4.6, 0.36 |  | -1.9 | -5.1, 1.2 |  |
| **Tumor Subsite** |  |  |  | 0.025 |  |  | 0.023 |
| Base of Tongue | 45 | — | — |  | — | — |  |
| Tonsil | 68 | -1.8 | -3.3, -0.23 |  | -1.8 | -3.4, -0.25 |  |
| **T Stage** |  |  |  | 0.001 |  |  |  |
| T1 | 53 | — | — |  |  |  |  |
| T2 | 58 | -0.21 | -1.7, 1.3 |  |  |  |  |
| T3 | 13 | 3.2 | 0.80, 5.6 |  |  |  |  |
| T4 | 20 | 3.1 | 1.0, 5.1 |  |  |  |  |
| **N Stage** |  |  |  | 0.052 |  |  |  |
| N0 | 17 | — | — |  |  |  |  |
| N1 | 88 | -1.2 | -3.3, 0.96 |  |  |  |  |
| N2 | 37 | 0.67 | -1.7, 3.0 |  |  |  |  |
| N3 | 3 | 3.0 | -2.1, 8.0 |  |  |  |  |
| **M Stage** |  |  |  | 0.9 |  |  |  |
| M1 | 4 | — | — |  |  |  |  |
| MX | 141 | -0.29 | -4.5, 3.9 |  |  |  |  |
| **Tumor Stage** |  |  |  | 0.008 |  |  |  |
| Stage I | 90 | — | — |  |  |  |  |
| Stage II | 30 | 2.7 | 1.1, 4.4 |  |  |  |  |
| Stage III | 10 | 2.1 | -0.58, 4.7 |  |  |  |  |
| Stage IV+ | 15 | 1.8 | -0.41, 4.0 |  |  |  |  |
| **Alcohol history** |  |  |  | >0.9 |  |  |  |
| No | 72 | — | — |  |  |  |  |
| Yes | 71 | 0.03 | -1.3, 1.4 |  |  |  |  |
| **Smoking history** |  |  |  | 0.3 |  |  |  |
| No | 64 | — | — |  |  |  |  |
| Yes | 83 | -0.70 | -2.1, 0.68 |  |  |  |  |
| No. Obs. |  |  |  |  | 113 |  |  |
| AIC |  |  |  |  | 644 |  |  |
| R² |  |  |  |  | 0.092 |  |  |
| ^1^CI = Confidence Interval | | | | | | | |

**Supplemental Table 12:** Univariable and multivariable analysis of PT score (Transportation SVI) (N=147)

|  | **Univariable** | | | | **Multivariable** | | |
| --- | --- | --- | --- | --- | --- | --- | --- |
| **Characteristic** | **N** | **Beta** | **95% CI**^1^ | **p-value** | **Beta** | **95% CI**^1^ | **p-value** |
| **Transportation SVI** | 147 | 0.31 | -2.1, 2.7 | 0.8 | -0.21 | -2.9, 2.5 | 0.875 |
| **Age at Diagnosis (Per 10 Years)** | 147 | 0.69 | -0.03, 1.4 | 0.061 |  |  |  |
| **Sex** |  |  |  | 0.11 |  |  | 0.367 |
| Female | 24 | — | — |  | — | — |  |
| Male | 123 | 1.5 | -0.35, 3.3 |  | 0.95 | -1.1, 3.0 |  |
| **Race** |  |  |  | 0.046 |  |  | 0.076 |
| Black/African American or Asian | 14 | — | — |  | — | — |  |
| White | 133 | -2.4 | -4.7, -0.05 |  | -2.3 | -4.9, 0.25 |  |
| **Insurance** |  |  |  | 0.040 |  |  |  |
| Medicare | 88 | — | — |  |  |  |  |
| Private | 55 | -1.6 | -3.1, -0.24 |  |  |  |  |
| Medicaid | 4 | -3.0 | -7.2, 1.2 |  |  |  |  |
| **p16 Status** |  |  |  | 0.093 |  |  | 0.179 |
| Negative | 12 | — | — |  | — | — |  |
| Positive | 135 | -2.1 | -4.6, 0.36 |  | -2.1 | -5.1, 0.96 |  |
| **Tumor Subsite** |  |  |  | 0.025 |  |  | 0.020 |
| Base of Tongue | 45 | — | — |  | — | — |  |
| Tonsil | 68 | -1.8 | -3.3, -0.23 |  | -1.8 | -3.4, -0.30 |  |
| **T Stage** |  |  |  | 0.001 |  |  |  |
| T1 | 53 | — | — |  |  |  |  |
| T2 | 58 | -0.21 | -1.7, 1.3 |  |  |  |  |
| T3 | 13 | 3.2 | 0.80, 5.6 |  |  |  |  |
| T4 | 20 | 3.1 | 1.0, 5.1 |  |  |  |  |
| **N Stage** |  |  |  | 0.052 |  |  |  |
| N0 | 17 | — | — |  |  |  |  |
| N1 | 88 | -1.2 | -3.3, 0.96 |  |  |  |  |
| N2 | 37 | 0.67 | -1.7, 3.0 |  |  |  |  |
| N3 | 3 | 3.0 | -2.1, 8.0 |  |  |  |  |
| **M Stage** |  |  |  | 0.9 |  |  |  |
| M1 | 4 | — | — |  |  |  |  |
| MX | 141 | -0.29 | -4.5, 3.9 |  |  |  |  |
| **Tumor Stage** |  |  |  | 0.008 |  |  |  |
| Stage I | 90 | — | — |  |  |  |  |
| Stage II | 30 | 2.7 | 1.1, 4.4 |  |  |  |  |
| Stage III | 10 | 2.1 | -0.58, 4.7 |  |  |  |  |
| Stage IV+ | 15 | 1.8 | -0.41, 4.0 |  |  |  |  |
| **Alcohol history** |  |  |  | >0.9 |  |  |  |
| No | 72 | — | — |  |  |  |  |
| Yes | 71 | 0.03 | -1.3, 1.4 |  |  |  |  |
| **Smoking history** |  |  |  | 0.3 |  |  |  |
| No | 64 | — | — |  |  |  |  |
| Yes | 83 | -0.70 | -2.1, 0.68 |  |  |  |  |
| No. Obs. |  |  |  |  | 113 |  |  |
| AIC |  |  |  |  | 644 |  |  |
| R² |  |  |  |  | 0.091 |  |  |
| ^1^CI = Confidence Interval | | | | | | | |
